# Serum C-peptide as a Predictor of Coronary Artery Disease in Middle-Aged Patients With Type 2 Diabetes Mellitus: A Cross-Sectional Analytical Study

**DOI:** 10.7759/cureus.74422

**Published:** 2024-11-25

**Authors:** Sarosh Kumar, Balakrishnan Valliyot

**Affiliations:** 1 Department of Internal Medicine, Government Medical College Kannur, Kannur, IND

**Keywords:** coronary artery disease, c-peptide, hyperinsulinemia, insulin resistance, type 2 diabetes mellitus

## Abstract

Introduction

Type 2 diabetes mellitus is a major public health problem. Coronary artery disease (CAD) is the major cause of morbidity and mortality due to diabetes. A subset of these patients develops this complication relatively early. Identifying such patients at increased risk of CAD using an appropriate marker is important in prevention. Insulin resistance plays an important role in the development and progression of atherosclerotic CAD. Measurement of serum C-peptide may help in identifying those with increased insulin resistance characterized by higher levels. This study was conducted to find the association between fasting C-peptide (FCP) levels and CAD in middle-aged patients with type 2 diabetes mellitus.

Materials and methods

This cross-sectional analytical study was done at the Medicine Outpatient Department of Government Medical College, Kannur, Kerala, South India. Type 2 diabetic patients aged 41-60 years were included. Patients on insulin, chronic liver disease, malignancy, critical illness, and an estimated glomerular filtration rate (eGFR) less than 45 ml/min/1.73 m^2^ were excluded. After a detailed clinical evaluation, blood samples were collected for FCP and other biochemical tests. A chemiluminescent immunoassay was used for measuring serum C-peptide levels. An independent t-test compared mean FCP levels between those with and without CAD, while binary logistic regression identified CAD risk factors and strength of association. The receiver operating characteristic (ROC) curve determined the cutoff value for predicting CAD.

Results

Among the 229 patients included, 50 (21.83%) had evidence of CAD. On an independent t-test, patients with CAD had significantly higher serum FCP levels (2.36 ± 0.80 vs. 1.86 ± 0.58 ng/ml, p < 0.001) than those without CAD. On binary logistic regression analysis, the association of serum FCP levels with CAD was found to be statistically significant (p = 0.001), with odds of CAD increasing by 2.98 times for each unit increase in FCP levels. On the ROC curve, FCP was found to have a good predictive ability with an area under curve (AUC) of 0.71 (0.61-0.80); using a cutoff value of FCP ≥ 2.13 ng/ml, the sensitivity, specificity, and accuracy for predicting CAD were 70%, 69%, and 69.2%, respectively. When patients with CAD were categorized based on quartiles of FCP levels, the majority of patients with CAD were in upper quartiles (Q3 and Q4) with higher serum C-peptide levels, further indicating an association between elevated C-peptide levels and CAD.

Conclusion

In this study, a significant positive association was observed between serum FCP levels and CAD in type 2 diabetic patients aged 41-60 years. The odds of CAD increased nearly threefold for each unit rise in serum FCP levels. Patients with a higher risk of CAD can be identified by using an optimal cutoff value of serum FCP. These findings underscore the potential role of FCP in early identification of patients at increased risk for CAD.

## Introduction

Type 2 diabetes mellitus is a major public health problem globally and is rapidly emerging in developing countries, including India. Coronary artery disease (CAD) is an important macrovascular complication and a major cause of morbidity and mortality due to diabetes [1]. CAD is becoming more prevalent in the middle-aged diabetic population due to changes in lifestyle practices [2]. Insulin resistance may play an important role in the development and progression of atherosclerotic CAD [3].

Patients with type 2 diabetes can develop both microvascular and macrovascular complications [4]. A subset of these patients develop CAD relatively early. Identifying such patients with an increased risk of developing CAD is vital in preventing this complication. Finding a marker for early identification of such patients will help in preventing this complication through aggressive lifestyle interventions, stringent glycemic control, and optimal control of other risk factors. C-peptide was once considered a biologically inactive marker of insulin production, but it is now recognized for its significant physiological functions. Research indicates that elevated circulating C-peptide levels in insulin-resistant patients may accumulate in the vessel walls and may promote atherogenesis [5]. Elevated levels of C-peptide may accelerate atherosclerosis in diabetic subjects through several mechanisms, including promoting inflammation, endothelial dysfunction, monocyte chemotaxis, and enhanced oxidative stress.

C-peptide is considered a better marker of endogenous insulin synthesis than insulin, and its serum levels depend on both insulin secretion and insulin resistance [6-8]. Measurement of serum C-peptide may help in identifying those with increased insulin resistance characterized by higher serum levels of C-peptide. Previous studies have shown some association between serum C-peptide levels and vascular complications in patients with type 2 diabetes; however, its specific association with CAD has yet to be clearly established in middle-aged diabetic patients. This study was conducted to find the association between serum fasting C-peptide (FCP) levels and CAD in middle-aged patients with type 2 diabetes mellitus.

## Materials and methods

This cross-sectional analytical study was done at the medicine outpatient department of Government Medical College, Kannur, Kerala, South India, from January 2023 to December 2023. Consecutive patients with type 2 diabetes mellitus diagnosed as per 2010 American Diabetes Association (ADA) criteria in the age group between 41 and 60 years with a duration of diabetes greater than five years and less than 12 years were included. Patients with critical illness, chronic liver disease, malignancy, and renal impairment with an estimated glomerular filtration rate (eGFR) less than 45 ml/min/1.73 m^2^ and on insulin treatment were excluded.

All participants underwent a thorough clinical examination, including a history of illnesses, oral antidiabetic medication details, and a detailed physical examination. Height, body weight, waist circumference, and blood pressure were measured, and body mass index (BMI) was calculated using the Quetelet Index and expressed as Kg/m^2^. Systemic hypertension was defined as per the American College of Cardiology/American Heart Association (ACC/AHA) Task Force on Clinical Practice Guidelines 2017 [9]. In this study, dyslipidemia was defined as per the 2013 ACC/AHA guideline on the treatment of blood cholesterol to reduce atherosclerotic cardiovascular risk in adults [10]. CAD was considered in patients who met at least one of the following criteria: records of a previous myocardial infarction, electrocardiogram findings typical of ischemia with positive cardiac biomarkers, a positive treadmill test, echocardiographic evidence of regional wall motion abnormalities, coronary angiographic evidence of CAD, or records of previous coronary artery bypass grafting or coronary angioplasty. All patients without established CAD were screened for CAD by electrocardiography, exercise stress test, and echocardiography. The eGFR was calculated using the chronic kidney disease-epidemiology collaboration (CKD-EPI) equation 2021-based calculator for glomerular filtration rate (GFR) using age, gender, and serum creatinine. After overnight fasting of a minimum of eight hours, blood samples were taken for fasting plasma glucose (FPG), FCP, fasting lipid profile, glycated hemoglobin (HbA1C), blood urea, serum creatinine, serum uric acid, serum sodium, and serum potassium. Serum C-peptide was measured using a chemiluminescent immunoassay for the quantitative determination of C-peptide levels. Homeostatic Model Assessment of Insulin Resistance (HOMA 2 IR) was calculated using the HOMA 2 calculator based on fasting plasma glucose and serum FCP.

The Institutional Ethics Committee (IEC) of Government Medical College Kannur, Pariyaram, Kerala, granted ethical approval for this research study with the reference number IEC No. 96/2019/GMCK. Written informed consent was obtained from all study participants before including in this research work.

Statistical analysis

The data collected were entered in MS Excel (Microsoft Corporation, Redmond, Washington, United States) and then analyzed using the IBM SPSS Statistics for Windows, Version 21 (Released 2012; IBM Corp., Armonk, New York, United States). Categorical variables were summarized as frequencies and percentages, and quantitative variables were expressed as mean and standard deviation. For comparison between groups, Pearson's chi-square test was used for categorical variables. An independent sample t-test was used for comparing the difference in the mean serum FCP levels between those patients with and without CAD. Multivariable binary logistic regression analysis was used to identify the risk factors and the strength of association for CAD. The receiver operating characteristic (ROC) curve was used to find the best cutoff value of serum FCP levels for identifying CAD. For comparison of HOMA 2 IR values between those patients with and without CAD, a Mann-Whitney U test was used as HOMA 2 IR values were not normally distributed and expressed as median and interquartile range (IQR). All statistical tests were two-tailed, with p ≤ 0.05 as the cutoff for statistical significance.

## Results

In this cross-sectional analytical study, 229 patients with type 2 diabetes mellitus were included. The mean age of the study group was 51.17 ± 5.66 years, and the mean duration of diabetes was 8.30 ± 1.84 years. Among them, 119 (52%) were males and 110 (48%) were females. In this group, 140 (61.1%) had systemic hypertension, and 155 (67.7%) had dyslipidemia as a comorbid illness. Among the study participants, 31 (13.5%) were current smokers, and 50 (21.8%) were regularly consuming alcohol. The mean BMI of them was 25.36 ± 2.96 kg/m^2^, and waist circumference (WC) was 90.75 ± 7.96 cm. The mean systolic blood pressure (SBP) and diastolic blood pressure (DBP) were 135.55 ± 11.44 and 83.14 ± 6.98 mm of Hg, respectively. The mean FCP levels of the study group were 1.97 ± 0.66 ng/ml. The median value of the participants' HOMA 2 IR was 1.62 with an IQR of 0.67 (1.28-1.95). The biochemical parameters of the study participants are presented in Table 1. 

**Table 1:** Biochemical parameters of the study participants (n = 229) FPG: fasting plasma glucose; PPG: prandial plasma glucose; HbA1C: glycated hemoglobin; Bld. urea: blood urea; sr. creatinine: serum creatinine; FCP: fasting C-peptide; T. chol: total cholesterol; TG: triglyceride; HDL: high-density cholesterol; LDL: low-density cholesterol; VLDL: very low-density cholesterol; eGFR: estimated glomerular filtration rate The data were represented as mean ± SD

Variables (reference range)	Mean ± SD	Variables (reference range)	Mean ± SD
FPG (<100 mg/dl)	143.59 ± 32.09	T. Chol (<200 mg/dl)	198.58 ± 25.10
PPG (<140 mg/dl)	229.84 ± 45.33	TG (<150 mg/dl)	137.21 ± 45.87
HbA1C (<5.7 %)	8.19 ± 1.26	HDL ( >40 mg/dl)	46.97 ± 7.40
Bld. urea ( 10-40 mg/dl)	26.02 ± 6.08	LDL ( <100 mg/dl)	123.15 ± 22.10
Sr. creatinine (0.6-1.2 mg/dl)	0.94 ± 0.18	VLDL (<30 mg/dl)	27.44 ± 8.99
FCP (ng/ml)	1.97 ± 0.66	eGFR (>90 ml/min/1.73 m^2^)	87.04 ± 17.46

Among this study group, 50 (21.83%) patients had evidence of CAD, and in 179 (78.17%) patients, there was no evidence of CAD. In those patients with CAD, 41 (82%) patients had coronary angiography-proven CAD, and in the rest, three (6%) had ST elevation myocardial infarction (STEMI), and six (12%) had non-ST elevation myocardial infarction (NSTEMI). In patients with CAG-proven CAD, six had single vessel disease, seven had double vessel disease, and 28 had triple vessel disease.

Among those with CAD, 37 (74%) were males and 13 (26%) were females. On chi-square analysis, a significant association was noted between CAD and gender (p < 0.001), with males at a higher risk with an odds ratio of 3.37 (95% CI: 1.68-6.76) as compared to females. In patients with CAD, 19 were smokers and 31 were nonsmokers, and on chi-square analysis, a significant association was noted between smoking and CAD (p < 0.001), with smokers at higher risk for CAD with an odds ratio of 8.53 (95% CI: 3.76 - 19.32) as compared to non-smokers. Among patients with CAD, 44 (88%) were hypertensive and 48 (96%) had dyslipidemia as comorbid illnesses. On chi-square analysis, a statistically significant association was observed between these comorbid illnesses and CAD. The results of the comparison of these categorical variables using chi-square testing are presented in Table 2. 

**Table 2:** Comparison of categorical variables between CAD and non-CAD patients HTN: systemic hypertension; DLP: dyslipidemia, χ^2^ value: chi-square value; CI: confidence interval Chi-square analysis of categorical variables of patients with and without CAD.

Variables	Category	CAD (n = 50)	Non-CAD (n = 179)	χ2 value	Odds ratio	95% CI	p-value
Sex	Males	37 (74%)	82 (45.81%)	12.44	3.37	(1.68-6.76)	0.001
	Females	13 (26%)	97 (54.19%)				
Smoking	Smoker	19 (38%)	12 (6.70%)	32.71	8.53	(3.76-19.32)	0.001
	Non-Smoker	31 (62%)	167 (93.30%)				
HTN	Yes	44 (88%)	96 (53.63%)	19.43	6.34	(2.57-15.63)	0.001
	No	6 (12%)	83 (46.37%)				
DLP	Yes	48 (96%)	107 (59.77%)	23.45	16.15	(3.81-68.55)	0.001
	No	2 (4%)	72 (40.23%)				

The mean age of patients with CAD was 53.52 ± 4.52 years, and those without CAD were 50.51 ± 5.78 years, and the association was found to be statistically significant (p < 0.001). The mean duration of diabetes for those with CAD was 8.52 ± 2.05 years and for those without CAD was 8.24 ± 1.78 years; no significant association was noted between the mean duration of diabetes and CAD. Patients with CAD had significantly higher mean serum creatinine values (0.99 ± 0.20 vs 0.92 ± 0.17 mg/dl) as compared to those without CAD (p-value = 0.015). Serum uric acid was significantly higher in patients with CAD (6.07 ± 0.83 vs. 5.59 ± 1.09 mg/dl) as compared to those without CAD (p-value = 0.004). No statistically significant differences were noted in the mean values of BMI, WC, FPG, HbA1C, eGFR, and lipid profile variables in those with and without CAD (Table 3). 

**Table 3:** Comparison of demographic and biochemical parameters between CAD and non-CAD patients BMI: body mass index; DM: diabetes mellitus; WC: waist circumference; FPG: fasting plasma glucose; T. cholesterol: total cholesterol; TG: triglyceride; HDL: high-density cholesterol; LDL: low-density cholesterol; Sr. creatinine: serum creatinine; Sr. uric acid: serum uric acid; eGFR: estimated glomerular filtration rate Independent sample T- test comparison of the mean values between CAD and non-CAD patients

Parameters	CAD (n = 50)	Non-CAD (n =179)	t-value	Mean difference	95% CI of mean difference	p-value
Age (years)	53.52 ± 4.52	50.51 ± 5.78	3.41	3.01	(1.27-4.76)	0.001
Duration of DM (yrs)	8.52 ± 2.05	8.24 ± 1.78	0.95	0.28	(- 0.30-0.86)	0.343
BMI (kg/m^2)^	25.18 ± 2.46	25.42 ± 3.09	- 0.49	-0.23	(-1.17-0.70)	0.622
WC (cm)	90.94 ± 7.54	90.69 ± 8.09	0.19	0.25	(-2.27-2.76)	0.847
FPG (mg/dl)	141.64 ± 29.84	144.13 ± 32.75	-0.48	-2.49	(-12.62-7.64)	0.629
HbA1C (%)	8.34 ± 1.12	8.14 ± 1.29	0.99	0.20	(-0.19-0.59)	0.319
T. cholesterol (mg/dl)	192.48 ± 28.06	200.28 ± 24.01	-1.96	-7.79	(-15.66-0.06)	0.052
TG (mg/dl)	144.52 ± 63.78	135.17 ± 39.44	1.28	9.35	(-5.09-23.79)	0.203
HDL (mg/dl)	46.04 ± 8.06	47.23 ± 7.21	-1.01	-1.19	(-3.53-1.14)	0.314
LDL (mg/dl)	117.28 ± 23.42	124.79 ± 21.50	-2.14	-7.51	(-14.42-0.59)	0.033
Blood urea (mg/dl)	26.60 8.33	25.85 ± 5.29	0.77	0.75	(-1.17-2.66)	0.444
Sr. creatinine (mg/dl)	0.99 ± 0.20	0.92 ± 0.17	2.46	0.70	(0.01 – 0.13)	0.015
Sr. uric acid (mg/dl)	6.07 ± 0.83	5.59 ± 1.09	2.89	0.17	(-0.15-0.80)	0.004
eGFR (mL/min/1.73 m^2^)	84.02 ± 17.09	87.88 ± 17.52	-1.39	-3.86	(-9.35-1.63)	0.167

In this study group, 172 (75.11%) patients were on metformin, 153 (66.81%) on sulfonyl ureas, 75 (32.75%) were on dipeptidyl peptidase-4 (DPP-4) inhibitors, 49 (21.40%) on sodium-glucose cotransporter-2 (SGLT-2) inhibitors, 11 (4.80%) on alpha-glucosidases inhibitors, and two (0.87%) on pioglitazone. Among patients with CAD, 38 (76%) were on metformin, 32 (64%) were on sulphonyl urea, 17 (34%) were on DPP-4 inhibitors, 15 (30%) on SGLT-2 inhibitors, and four (8%) were on alpha-glucosidases inhibitors. In the non-CAD group, 134 (74.86%) were taking metformin, 121 (67.60%) were on sulphonyl urea, 58 (32.40%) were on DPP-4 inhibitors, 34 (18.99%) on SGLT-2 inhibitors, seven (3.91%) were on alpha-glucosidases inhibitors, and two patients were on pioglitazone. The oral antidiabetic drug exposure among the CAD group and non-CAD group were almost similar, and on chi-square analysis, no significant association was observed for each class of oral antidiabetic drug and CAD status.

**Figure 1:**
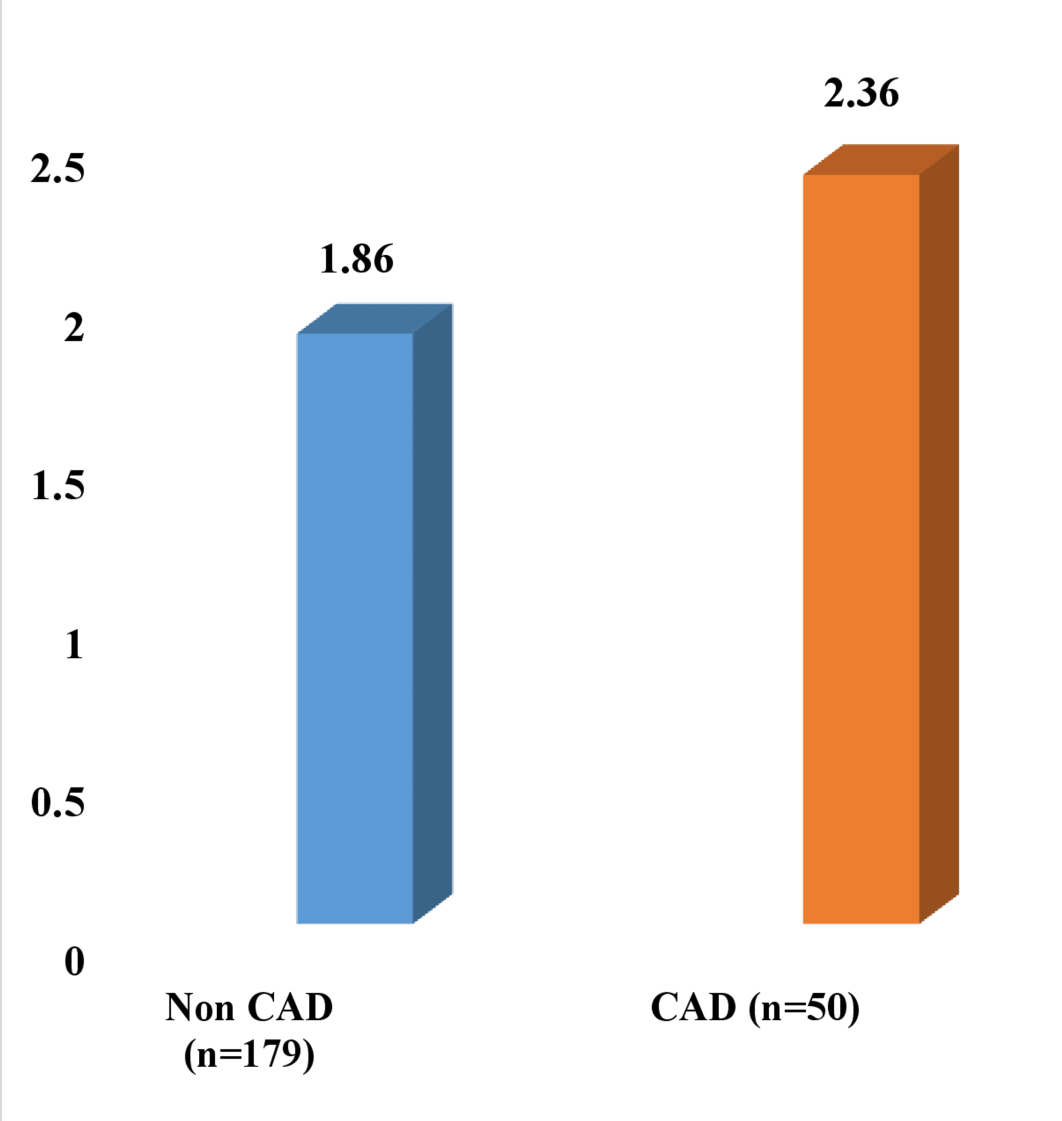
Independent sample t-test result comparing the mean fasting C-peptide levels of patients with and without CAD Mean serum fasting C-peptide levels of 2.36 ± 0.80 ng/ml for patients with CAD as compared to 1.86 ± 0.58 ng/ml for patients without CAD

An independent sample t-test was conducted to find the association between CAD and serum FCP levels. The patients with CAD had significantly higher mean serum FCP levels (2.36 ± 0.80 vs. 1.86 ± 0.58 ng/ml) than those without CAD (t-value 4.97, mean difference 0.49 (95% confidence interval 0.29-0.69), p = 0.001) (Figure 1).

The association between CAD and HOMA 2 IR values was analyzed using the Mann-Whitney U test, as the distribution of HOMA 2 IR values in both groups was not following normal distribution. The median HOMA 2 IR level was significantly higher in the CAD group (median (IQR): 1.97 (1.48-2.46)) as compared to the non-CAD group (median (IQR): 1.55 (1.26-1.85)). The Mann-Whitney U test showed a statistically significant difference in HOMA 2 IR values between the groups (U = 2678.50, Z = 4.34, p < 0.001).

The distribution of patients with CAD was analyzed based on quartiles of serum FCP levels and HOMA 2 IR values. The distribution of CAD patients quartile-wise was almost similar for FCP levels and HOMA 2 IR values. It was noted that most of the patients belonged to the fourth quartile, with the highest FCP and HOMA IR 2 values, and the majority of patients belonged to combined third and fourth quartiles (Q3 and Q4) with higher FCP and HOMA 2 IR values (Table 5).

**Table 4:** Distribution of patients with and without CAD based on quartiles of serum fasting C-peptide and HOMA 2 IR levels CAD: coronary artery disease; HOMA 2 IR: Homeostatic Model Assessment of Insulin Resistance Fasting C-peptide: Q1-first quartile equal to or less than 1.53 ng/ml, Q2-second quartile with levels above 1.53 ng/ml and below 1.95 ng/ml, Q3-third quartile with levels above 1.95 ng/ml and below 2.32ng/ml, and Q4-fourth quartile with levels above 2.32 ng/ml HOMA 2 IR: Q1-first quartile equal to or less than 1.28, Q2-second quartile with levels above 1.28 and below 1.62, Q3-third quartile with levels above 1.62 and below 1.95, and Q4-fourth quartile with levels above 1.95

Fasting C-peptide	Q1 (0.40, 1.53)	Q2 (1.53, 1.95)	Q3 (1.95, 2.32)	Q4 (2.32, 3.65)
CAD (n = 50)	9 (18%)	5 (10%)	9 (18%)	27 (54%)
Non-CAD (n = 179)	48 (26.8%)	54 (30.2%)	47 (26.3%)	30 (16.7%)
HOMA 2 IR	Q1 [0.35, 1.28)	Q2 (1.28, 1.62)	Q3 (1.62, 1.95)	Q4 (1.95, 3.37)
CAD (n = 50)	9 (18%)	6 (12%)	9 (18%)	26 (52%)
Non-CAD (n = 179)	48 (26.8%)	51 (28.5%)	49 (27.4%)	31 (17.3%)

A multivariable binary logistic regression analysis was performed to determine the effects of risk factors on the likelihood of having CAD. Male sex, systemic hypertension, dyslipidemia, smoking, and FCP were found significant and associated with higher odds of developing CAD. The association with FCP levels was highly significant (p = 0.001), with the odds of developing CAD increasing 2.986 times for each unit (ng/ml) increase in FCP levels, indicating a strong association (Table 6). 

**Table 5:** Binary logistic regression analysis of factors of coronary artery disease in patients with type 2 diabetes mellitus B: coefficient; SE: standard error; Wald: Wald statistic; Exp (B): exponentiated B coefficient (odds ratio); CI: confidence interval. Logistic regression analysis of coronary artery disease in middle aged patients with type 2 diabetes mellitus; dura of DM: duration of type 2 diabetes mellitus in years, HTN: systemic hypertension; DLP: dyslipidemia; BMI: body mass index in Kg/m2; eGFR: estimated glomerular filtration rate in ml/min/1.73 m^2;^ HbA1c: glycated hemoglobin in %; FCP: serum fasting C-peptide levels in ng/ml *Statistically significant p-value (p < 0.05). Male sex, systemic hypertension, dyslipidemia, and serum fasting C-peptide levels were found statistically significant

Variables	B	S. E	Wald	Exp (B)	95 % C.I.	p-value
Age (years)	0.014	0.047	0.092	1.014	(0.926 – 1.111)	0.762
Dura of DM	-0.170	0.140	1.493	0.843	(0.641 – 1.109)	0.222
Male Sex	1.081	0.492	4.820	2.947	(1.123 – 7.735)	0.028^*^
HTN	1.493	0.561	7.089	4.449	(1.483 – 13.351)	0.008^*^
DLP	1.893	0.789	5.760	6.641	(1.415 – 31.171)	0.016^*^
Smoking	1.314	0.555	5.616	3.722	(1.255 – 11.035)	0.018^*^
BMI	-0.137	0.086	2.527	0.872	(0.736 – 1.033)	0.112
eGFR	-0.019	0.015	1.654	0.981	(0.952 – 1.010)	0.198
HbA1c	0.186	0.191	0.944	1.204	(0.828 – 1.753)	0.331
FCP	1.094	0.328	11.11	2.986	(1.569 – 5.682)	0.001^*^

**Figure 2:**
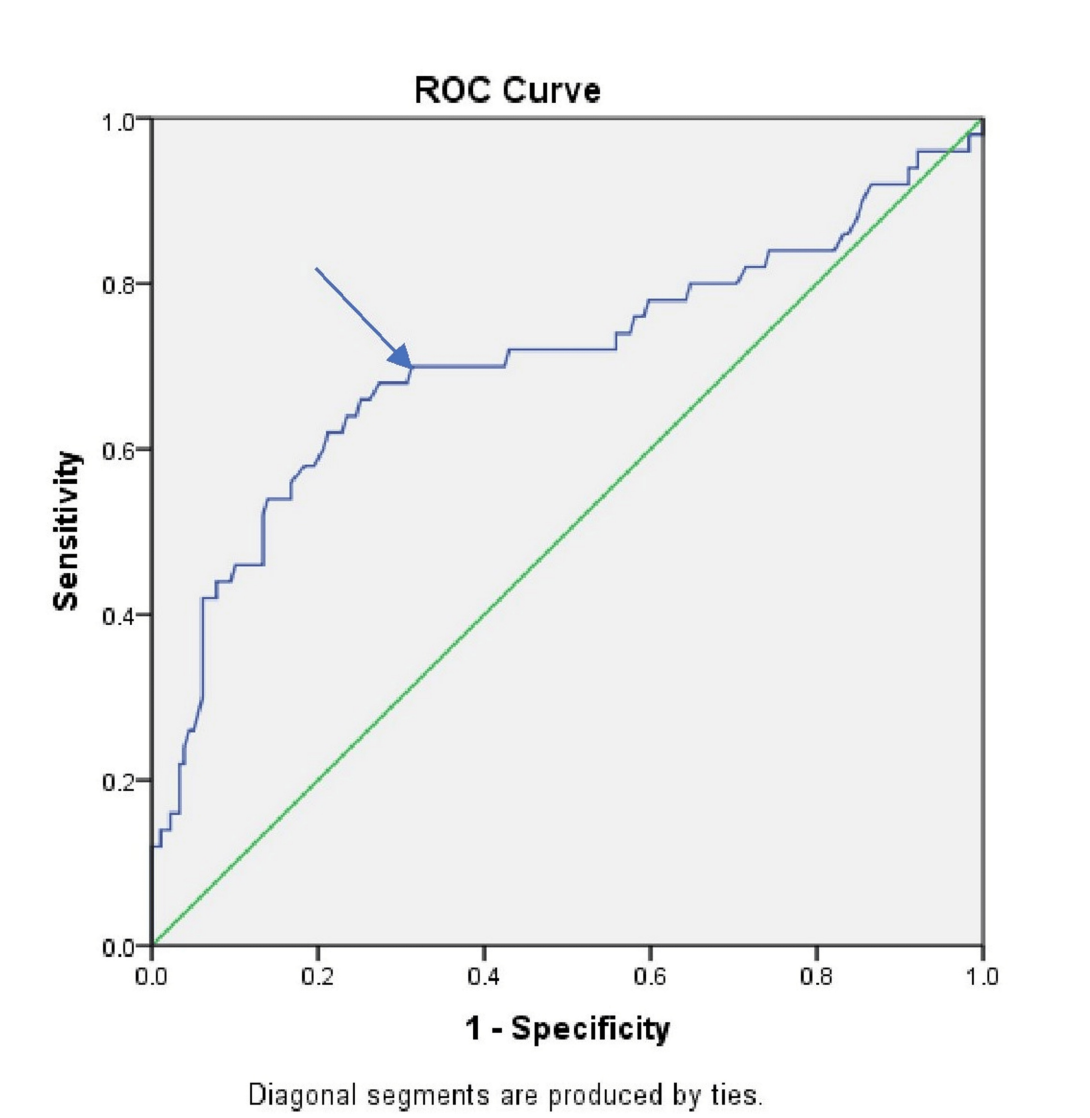
ROC curve of serum fasting C-peptide in predicting coronary artery disease ROC curve: receiver operating characteristic curve; sensitivity: true positive rate (TPR) on the y-axis; 1-specificity: false positive rate (FPR) on the x-axis ROC curve analysis in predicting CAD based on fasting C-peptide levels with a cutoff value of 2.13 ng/ml; the sensitivity and specificity were 70% and 69%, respectively. Area under curve (AUC) was 0.71 (95%CI: 0.61 - 0.80) and significance (p-value) of 0.001. The arrow indicates the cutoff point of this sensitivity and specificity

ROC curve analysis showed that FCP levels have a good predictive ability to distinguish CAD from non-CAD subjects, with an area under the curve of 0.71 (95% CI: 0.61-0.80) and a p-value of 0.001. Using a cutoff value of FCP ≥ 2.13 ng/ml, the sensitivity and specificity for predicting CAD were 70% and 69%, respectively. The positive predictive value, negative predictive value, and accuracy were 38.7%, 89.2%, and 69.2%, respectively (Figure 2). 

## Discussion

In this cross-sectional analytical study, we aimed to find the association between serum FCP levels and CAD in patients with type 2 diabetes in the age group of 41-60 years. In this study, we found a significant positive association between serum FCP levels and CAD. Patients with CAD had significantly higher mean FCP levels than those without CAD on independent sample t-test. On logistic regression analysis, FCP was found as an independent factor associated with CAD, and the odds of developing CAD increased by 2.98 times for each unit increase in FCP levels. ROC curve analysis revealed a good diagnostic value for FCP in predicting CAD with good sensitivity and high negative predictive value. When patients with CAD were categorized based on quartiles of FCP levels, the majority of patients were in the higher quartiles (Q3 and Q4) with higher serum C-peptide levels, further indicating an association between elevated C-peptide levels and CAD.

In this study of the middle-aged diabetic population, cardiovascular risk factors such as age, male sex, smoking, systemic hypertension, and dyslipidemia were found to be significantly associated with CAD. No statistically significant difference was noted in the mean duration of diabetes, BMI, and HbA1C between these two groups. No significant difference was observed between the CAD and non-CAD groups with regard to antidiabetic drug exposure to influence the serum FCP levels. Both metformin and sulfonylureas exposures were almost similar in both groups, and there was no statistical significance. With regard to the cardiovascular risk modification due to antidiabetic drugs, no significant difference was observed in the use of SGLT-2 inhibitors among the two groups, and none of the study patients were using oral or injectable GLP-1 receptor agonists.

HOMA 2 IR values were found to be significantly higher in the CAD group as compared to the non-CAD group on the Mann-Whitney U test. When patients with CAD were categorized based on quartiles of HOMA 2 IR values like FCP values, the majority of patients were in the higher quartiles (Q3 and Q4) with higher HOMA 2 IR values. The distribution of patients with CAD in each quartile of HOMA 2 IR was almost similar to FCP. As HOMA 2 IR also takes into account corresponding fasting plasma glucose, more studies may be needed to confirm whether FCP measurement may be as good as HOMA 2 IR in CAD risk prediction. 

Some previous studies have investigated the relationship between serum C-peptide levels and CAD in patients with type 2 diabetes mellitus. While some studies have demonstrated a positive association between elevated serum C-peptide levels and the risk of CAD, few have reported a lack of significant association. Our results were in concordance with previous studies that have reported a positive association between C-peptide levels and CAD. The study by Wang et al. done in Shandong University, China, demonstrated that higher serum C-peptide levels were associated with an increased risk of CAD in patients with type 2 diabetes [11]. Yan et al. reported the effect of C-peptide on cardiovascular risk as bidirectional, with a beneficial role at lower levels and a detrimental role at a higher level in nondiabetic adults and patients with newly diagnosed type 2 diabetes [12]. Harnishsingh et al., in an observational study, evaluated the role of C-peptide and CAD; they reported that higher C-peptide levels appeared to correlate well with the presence and severity of CAD [13].

The association between elevated C-peptide levels and CAD observed in this study may indicate an increased risk of atherosclerotic CAD due to heightened insulin resistance in these individuals. Higher serum C-peptide levels likely reflect the increased insulin secretion due to increased insulin resistance. More experimental studies are needed to prove the role of elevated levels of C-peptide and insulin directly acting in the development and progression of atherosclerotic CAD. In the clinical management of patients with type 2 diabetes, our findings support the consideration of serum C-peptide as a marker for cardiovascular risk stratification. Measuring C-peptide levels could provide insight into their risk for developing CAD, like the traditional risk factors. This could have implications for management by providing more aggressive cardiovascular prevention strategies in those patients with elevated C-peptide levels.

Further studies from different regions and populations are required to conclusively establish the association between FCP levels and CAD. We recommend measuring FCP levels as a routine initial test following diagnosis, as it aids in cardiovascular risk stratification. Newer measurement techniques of serum C-peptide are more reliable, consistent, and affordable, enhancing the test’s effectiveness. The main limitation of our study is the cross-sectional design, which limits the ability to establish a causal relationship between C-peptide levels and CAD, and prospective studies are needed to confirm this causal association.

## Conclusions

The study observed a significant association between elevated serum FCP levels and an increased risk of CAD in middle-aged patients with type 2 diabetes. For each unit increase in FCP levels, the odds of CAD nearly tripled. Using an optimal cutoff value, CAD can be predicted with good sensitivity and specificity. These findings underscore the potential role of FCP in the early identification of patients at increased risk for CAD.
